# Supplementary material for: Enjoyment of Physical Activity—Not MVPA during Physical Education—Predicts Future MVPA Participation and Sport Self-Concept
Source: Sports (Basel). 2021 Sep 10;9(9):128. doi: 10.3390/sports9090128 (PMC8470923; doi:10.3390/sports9090128)
Supplement: Supplementary file 1 [file sports-09-00128-s001.zip › sports-1344019-supplementary.pdf]

sports

MDPI

Article

Enjoyment of Physical Activity—Not MVPA during Physical Education—Predicts Future MVPA Participation and Sport Self-Concept

Jared D. Ramer [1]*, Natalie E. Houser [2], Robert J. Duncan [3], and Eduardo E. Bustamante [1]

[1] College of Applied Health Sciences, University of Illinois at Chicago, Chicago, IL 60607, USA, ebusta2@uic.edu
[2] College of Kinesiology, University of Saskatchewan, Saskatoon S7N 5B2, Canada; natalie.houser@usask.ca
[3] College of Health and Human Sciences, Purdue University, West Lafayette, IN 47907, USA; duncan99@purdue.edu
* Correspondence: jramer2@uic.edu

Citation: Ramer J. D., Houser N. E., Duncan R. J., Bustamante E. E. Enjoyment of Physical Activity—not MVPA during Physical Education—Predicts Future MVPA Participation and Sport Self–Concept. *Sports* **2021**, *9*, 128. https://doi.org/10.3390/sports9090128

Academic Editor: Daniel Almeida Marinho

Received: 31 July 2021
Accepted: 6 September 2021
Published: 10 September 2021

Publisher's Note: MDPI stays neutral with regard to jurisdictional claims in published maps and institutional affiliations.

Copyright: © 2021 by the authors. Submitted for possible open access publication under the terms and conditions of the Creative Commons Attribution (CC BY) license (https://creativecommons.org/licenses/by/4.0/).

Table S1. Correlation Matrix of All Study Variables.

	G5 Enjoy PA	G5 PE MVPA	G5 PE % MVPA	G5 PE Minutes/week	G5 Weekday MVPA	G6 Sport Self-Concept	G6 Enjoy PA	G6 MVPA All Days	G6 BMI	G5 BMI	Sex	Limited Physical Functioning
G5 Enjoy PA	–											
G5 PE MVPA	0.057	–										
G5 PE % MVPA	0.065	0.633 ***	–									
G5 PE Minutes/week	0.011	0.746 ***	0.054	–								
G5 Weekday MVPA	0.184 ***	0.074 *	0.060	0.045	–							
G6 Sport Self−Concept	0.351 ***	0.057	0.080 *	0.001	0.120 ***	–						
G6 Enjoy PA	0.034	−0.007	−0.025	0.008	0.020	0.075 *	–					
G6 MVPA All Days	0.194 ***	0.050	0.094 *	0.002	0.407 ***	0.185 ***	0.198 ***	–				
G6 BMI	−0.141 ***	−0.101 **	−0.062	−0.078 *	−0.146 ***	−0.011	−0.021	−0.097 *	–			
G5 BMI	−0.135 ***	−0.087 *	−0.055	−0.062	−0.148 ***	−0.003	−0.156 ***	−0.111 **	0.946 ***	–		
Sex	0.021	−0.095 **	−0.083 *	−0.045	−0.214 ***	−0.085 **	−0.035	−0.272 ***	−0.043	−0.057	–	
Limited Physical Functioning	−0.058	0.006	0.004	−0.005	0.003	−0.021	0.012	−0.017	0.064	0.056	0.010	–

Pearson Correlation. * $p < .05$, ** $p < .01$, *** $p < .001$.

Table S2. Unstandardized and Standardized Estimates Within Structural Equation Model.

**Interaction**		**Estimate**	**S.E.**	**C.R.**	**P−value**	**β**
**Independent Variables**						
G5 Enjoy PA−>	G5 Weekday MVPA	8.452	1.626	5.198	***	0.173
	G6 Sport Self−Concept	0.498	0.045	11.121	***	0.347
	G6 Enjoy PA	0.558	0.03	18.61	***	0.538
	G6 MVPA All Days	5.739	1.611	3.563	***	0.125
	G6 BMI	−0.071	0.074	−0.971	0.332	−0.012
PE MVPA minutes/week −>	G5 Weekday MVPA	0.081	0.065	1.244	0.213	0.044
	G6 Sport Self−Concept	0.002	0.002	1.058	0.29	0.035
	G6 Enjoy PA	0.000	0.001	.251	0.802	0.008
	G6 MVPA All Days	−0.011	0.063	−0.169	0.866	−0.006
	G6 BMI	−0.004	0.003	−1.491	0.136	−0.019
Mediator						
G5 PAM weekday MVPA −>	G6 Sport Self−Concept	0.001	0.001	1.085	0.278	0.037
	G6 Enjoy PA	0.000	0.001	0.558	0.577	0.018
	G6 MVPA All Days	0.317	0.034	9.181	***	0.338
	G6 BMI	0.000	0.002	0.017	0.987	0.000
Control						
G5 BMI −>	G5 Weekday MVPA	−1.171	0.32	−3.659	***	−0.129
	G6 Sport Self−Concept	0.015	0.009	1.707	0.088	0.056
	G6 Enjoy PA	−0.018	0.006	−3.04	0.002	−0.093
	G6 MVPA All Days	−0.385	0.312	−1.234	0.217	−0.045
	G6 BMI	1.044	0.013	78.535	***	0.943
Sex −>	G5 Weekday MVPA	−18.128	2.65	−6.84	***	−0.223
	G6 Sport Self−Concept	−0.185	0.073	−2.537	0.011	−0.078
	G6 Enjoy PA	−0.089	0.05	−1.791	0.073	−0.052
	G6 MVPA All Days	−15.912	2.645	−6.015	***	−0.209
	G6 BMI	0.18	0.12	1.497	0.134	0.018
Limited Physical Function −>	G5 Weekday MVPA	1.497	3.225	0.464	0.642	0.015
	G6 Sport Self−Concept	−0.023	0.086	−0.27	0.787	−0.008
	G6 Enjoy PA	0.042	0.058	0.719	0.472	0.02
	G6 MVPA All Days	−0.717	3.109	−0.23	0.818	−0.008
	G6 BMI	−0.027	0.141	−0.189	0.85	−0.002
Covariances						
Interactions		Estimate	S.E.	C.R.	P−value	Corr.
G5 Enjoy <−> PE MVPA minutes/week		1.078	0.638	1.69	0.091	0.059
G5 Enjoy <−> G5 BMI		−0.51	0.128	−3.979	***	−0.138
G5 Enjoy <−> Sex		0.01	0.013	0.765	0.445	0.024
G5 Enjoy <−> Limited Physical Functioning		−0.016	0.011	−1.459	0.144	−0.047
PE MVPA minutes/week <−> G5 BMI		−9.071	3.592	−2.525	0.012	−0.092
PE MVPA minutes/week <−> Sex		−1.07	0.373	−2.866	0.004	−0.097
PE MVPA minutes/week <−> Limited Physical Functioning		0.051	3.16	0.161	0.872	0.006
Sport Self−Concept residual <−> G6 Enjoy PA residual		0.229	0.027	8.363	***	0.292
Sport Self−Concept residual <−< G6 MVPA All Days		3.566	1.359	2.625	0.009	0.096

Standard Error (S.E.), Critical Ratio (C.R.), Correlation (Corr.).

Table S3. Model Total Effects.

	G5 Enjoy PA	G5 PE MVPA	G5 Weekday MVPA	G5 BMI	Sex	Limited Physical Functioning
G5 Weekday MVPA	8.452(0.173)	0.081(0.044)	–	−1.171(0.129)	−18.128(0.223)	1.497(0.015)
G6 Sports Self−Concept	0.507(0.353)	0.002(0.036)	0.001(0.037)	0.014(0.051)	−0.205(0.086)	−0.022(0.008)
G6 Enjoy PA	0.561(0.541)	0.000(0.008)	0.000(0.018)	−0.018(0.096)	−0.096(0.056)	0.043(0.021)
G6 MVPA All Days	8.415(0.184)	0.015(0.009)	0.317(0.338)	−0.756(0.089)	−21.651(0.285)	−0.243(0.003)
G6 BMI	−0.071(0.012)	−0.004(0.019)	0.000(0.000)	1.044(0.943)	0.18(0.018)	−0.027(0.002)

Table S4. Model Direct Effects.

	G5 Enjoy PA	G5 PE MVPA	G5 Weekday MVPA	G5 BMI	Sex	Limited Physical Functioning
G5 Weekday MVPA	8.452(0.173)	0.081(0.044)	–	−1.171(0.129)	−18.128(0.223)	1.497(0.015)
G6 Sports Self−Concept	0.498(0.347)	0.002(0.035)	0.001(0.037)	0.015(0.056)	−0.185(0.078)	−0.023(0.008)
G6 Enjoy PA	0.558(0.538)	0.000(0.008)	0.000(0.018)	−0.018(0.093)	−0.089(0.052)	0.042(0.02)
G6 MVPA All Days	5.739(0.125)	−0.011(0.006)	0.317(0.338)	−0.385(0.045)	−15.912(0.209)	−0.717(0.008)
G6 BMI	−0.071(0.012)	−0.004(0.019)	0.000(0.000)	1.044(0.943)	0.18(0.018)	−0.027(0.002)

Table S5. Model Indirect Effects.

	G5 Enjoy PA	G5 PE MVPA	G5 Weekday MVPA	G5 BMI	Sex	Limited Physical Functioning
G5 Weekday MVPA	0.000(0.000)	0.000(0.000)	–	0.000(0.000)	0.000(0.000)	0.000(0.000)
G6 Sports Self−Concept	0.009(0.006)	0.000(0.002)	0.000(0.000)	−0.001(0.005)	−0.02(0.008)	0.002(0.001)
G6 Enjoy PA	0.003(0.003)	0.000(0.001)	0.000(0.000)	0.000(0.002)	−0.007(0.004)	0.001(0.003)
G6 MVPA All Days	2.676(0.058)	0.026(0.015)	0.000(0.000)	−0.371(0.044)	−5.739(0.076)	0.474(0.005)
G6 BMI	0.000(0.000)	0.000(0.000)	0.000(0.000)	0.000(0.000)	0.000(0.000)	0.000(0.000)
